# Xylogenesis under future climates: enhanced growth of balsam fir in a warming boreal forest

**DOI:** 10.3389/fpls.2025.1563051

**Published:** 2025-07-10

**Authors:** Minhui He, Jean-Daniel Sylvain, Roberto Silvestro, Guillaume Drolet, Richard Arsenault, Sergio Rossi

**Affiliations:** ^1^ Laboratoire sur les écosystèmes terrestres boréaux, Département des Sciences Fondamentales, Université du Québec à Chicoutimi, Chicoutimi, QC, Canada; ^2^ Centre de Géomatique du Québec, Chicoutimi, QC, Canada; ^3^ Direction de la recherche forestière, Ministère des Ressources naturelles et des Forêts, Québec, QC, Canada; ^4^ Hydrology, Climate and Climate Change Laboratory, École de Technologie Supérieure, Montréal, QC, Canada

**Keywords:** cambial activity, cell enlargement, secondary wall thickening, cell production, warming trend

## Abstract

Understanding the effects of climate variability on tree growth is crucial to predict forest carbon sequestration under global climate change. This study investigates the dynamics of wood formation in balsam fir in response to historical climatic data and projected variations. Weekly microcores were collected from 33 permanent plots in a boreal forest in Québec, Canada, over five growing seasons from 2018 to 2022. Warmer spring temperatures were associated with earlier cell enlargement initiation and increased cell production, whereas precipitation exerted a limited effect. An advancement in the onset of cell enlargement by 14–42 days and a 25–85% increase in cell production from 2051–2080 relative to the reference period (1981–2010) were predicted. Our results reveal potential shifts in growth dynamics and xylem production that could modify the growth processes in balsam fir, including carbon sequestration in the boreal forest ecosystems of Eastern Canada.

## 1 Introduction

Climate change is one of the most pressing environmental challenges of the 21^st^ century, with profound impacts on terrestrial ecosystems worldwide (Higgins et al., 2023; Li et al., 2018; Nolan et al., 2018). The boreal forest, the largest terrestrial biomes of Earth, plays a critical role in carbon sequestration and global climate regulation (Astrup et al., 2018; Gauthier et al., 2015; Kolomyts, 2022; Pan et al., 2024). However, this forest biome is facing unprecedented challenges due to global climate change (Gauthier et al., 2014; Metsaranta et al., 2023; Price et al., 2013). Compared to other forest ecosystems, the boreal forest is expected to experience the greatest warming, which characterizes the regions at the higher latitudes (Gauthier et al., 2015). Climate projections predict an increase by up to 30% in precipitation during winter, spring, and autumn in southeastern Canada (Pierce et al., 2023). These shifts in temperature and precipitations patterns may affect the timings of the growing season and forest productivity (D’Orangeville et al., 2018; Silvestro et al., 2024), modify composition and structure of the stands, finally altering the distribution and abundance of species across the landscape (Klipel et al., 2022; Pardi et al., 2024). These variations in species composition could in turn impact ecosystem dynamics, including changes in nutrient cycling, habitat suitability and biodiversity (Bledsoe and Ernest, 2019; Rocha et al., 2018). Therefore, a comprehensive understanding of tree growth dynamics and their relationship with climate variables is essential for anticipating the impacts of environmental changes on boreal ecosystems.

Balsam fir [*Abies balsamea* (L.) Mill.] is one of the most abundant softwood species in the boreal forest of eastern Canada. For this reason, any impact of a changing climate on growth and productivity of balsam fir may have extensive ecological and economic consequences. Several studies have investigated the sensitivity of this species to weather conditions and disentangled the eco-physiological traits influencing its growth (Desponts et al., 2011; Duchesne et al., 2012). Wood formation dynamics (i.e., xylogenesis), plays a crucial role in the growth and development of trees, influencing forest dynamics and ecosystem services. A considerable number of studies have focused on the phenological pattern of balsam fir xylogenesis employing either microcores (Deslauriers et al., 2003a; Deslauriers and Morin, 2005; D’Orangeville et al., 2013a; Krause et al., 2010; Silvestro et al., 2023) or dendrometers (Duchesne and Houle, 2011; Tardif et al., 2001). These studies indicated that changes in temperature, at either seasonal or interannual scales, influence the timings and duration of xylogenesis. Warmer temperatures during the growing season can speed up wood formation by influencing the rate of cell production and development, and ultimately shaping the growth patterns in the tree ring (Chen et al., 2019; Rossi et al., 2016, 2008). Concurrently, a decrease in soil water content was linked to drops in cell division, delayed enlargements of tracheids, smaller cells with thicker secondary walls (D’Orangeville et al., 2013b) and overall, a reduced number of xylem cells produced (Deslauriers et al., 2003b).

Despite of the extensive literatures on balsam fir, our understanding of how environmental factors influence xylem phenology remains incomplete. The effects of temperature shifts on wood phenology and growth performances likely depend on the seasons in which they occur and may also interact with precipitations and other local environmental factors (Collier et al., 2024; Vaughn et al., 2022, 2021). Achieving a comprehensive understanding of the responses of balsam fir to climate is further complicated by the phenological variability among trees within a population (Silvestro et al., 2022). The complexity of these processes underscores the need for ongoing wood phenology research, that incorporates data from a representative number of trees to predict the evolution of growth under climate change scenarios.

This study investigated the interaction between xylem phenology, cell production and climate conditions, by weekly microcoring 160 trees during the growing seasons between 2018 to 2022 in a balsam fir stand in southeastern Canada. We analyzed the relationships between climate and xylem phenology using monthly and seasonal minimum and maximum temperatures and precipitations. These analyses were extended into predictive modeling to map xylogenesis under current and future climate scenarios to provide an overview of how the ongoing climate change could reshape the growth patterns of balsam fir in Québec. We tested the hypothesis that warming (1) results in an earlier onset of xylogenesis and enhanced cell production, and (2) has a stronger impact on growth reactivation compared with growth cessation.

## 2 Materials and methods

### 2.1 Study area

The study area is in stands dominated by 25-35-year-old balsam fir in the Forêt Montmorency, Québec, Canada (47.3°N, 71.1°W), within the balsam fir–white birch bioclimatic domain (Silvestro et al., 2022). The experimental forest originated from clearcuts realized in 1993–1994 to assess the impact of harvesting on peak flow in boreal conditions. The climate is continental with long, cold winters and a short growing season characterized by cool temperatures and abundant precipitations. January is the coldest month with a mean minimum temperature of -21.6°C, and July is the warmest with a mean maximum temperature of 21.1°C during the period 1981–2022. Monthly minimum and maximum temperatures > 0°C normally occur during June–September and April–October, respectively. The annual precipitation reaches 1464 mm, 26% of which falling during June–August. An increased evapotranspiration is resulting in a decrease in water availability during recent summers (Isabelle et al., 2021).

### 2.2 Sampling and data collection

A total of 33 permanent plots measuring 20 m × 20 m were established to examine the xylogenesis of balsam fir. For each year, 4–5 codominant individuals were selected within each plot, resulting in at least 160 sampled trees per year (**Supplementary Table S1**). Trees with polycormic stems, partially dead crowns, reaction wood, or obvious damage from parasites were excluded from the selection. We collected microcores weekly at 1–3 m along the stem from April to October during 2018–2022 using a Trephor (Rossi et al., 2006a).

The microcores were dehydrated through successive immersions in ethanol and d-limonene and embedded in paraffin. Cross sections of 8 µm thickness were cut with a rotary microtome, stained with cresyl violet acetate (Rossi et al., 2006b) and observed under the microscope. The different phases of xylogenesis were identified under visible and polarized light at magnifications of × 400. Cells were counted across three radial rows and classified in the phase of cambium, enlargement, wall-thickening, and lignification, or mature (Deslauriers et al., 2003a). Cambial cells had thin cell walls and small radial diameters. Enlarging cells had a large radial diameter, at least twice that of a cambial cell, but lacked glistening under polarized light. Wall-thickening cells glistened under polarized light. The lignification stained the cell walls from violet to blue. The change in color over the whole secondary cell wall revealed the ending of lignification and maturity of the cell (Rossi et al., 2006b). The beginning of xylem formation was defined when at least one enlarging cell was observed in spring. In autumn, the absence of cells undergoing wall thickening and lignification indicated the ending of cell differentiation. The onset and ending of each cell developmental phase were expressed as the day of year (DOY).

### 2.3 Climate data

Climate data was recorded by the Canadian meteorological service at 3.5 km from the study area (47°19′22′′ N, 71°08′54′′ W; altitude 672.8 m). Missing values (accounting for 67%) occurred in the years 1999–2004, so the longer-term reanalysis dataset of ERA5 was used (Hersbach et al., 2020). Daily temperature and total precipitation were downloaded using the nearest grid point. The performance of the reanalyzed data was evaluated by the series recorded by the local weather station using the Pearson correlation. Daily values were converted in monthly, seasonal, and annual data, and used for the successive analyses. We quantified trends in climate conditions using linear regression models evaluated with Mann-Kendall tests.

Mapping of xylogenesis for the future period was based on the predicted mean temperature of all model members from the Coupled Model Intercomparison Project (CMIP6). Two socio-economic pathways were selected: ssp126 and ssp585. The temperatures were extracted for 1981–2010 and 2051–2080 over a spatial region of 40–65°N, 85–55°E, representing the distribution of balsam fir in Quebec. The model temperatures were first downscaled to the same resolution as the ERA5 reanalysis grid using a bilinear interpolation scheme. The grid points were bias-corrected using a delta-based approach on a monthly scale. ERA5 was used as a reference for the period 1981–2010. The differences between ERA5 and model data were computed for each month and then applied to the future model data (2051–2080), generating bias-corrected temperature for each location. This was repeated for each climate model and socioeconomic pathway, and the overall average of all models was taken to obtain a robust estimation of reference period and future period temperatures over the study domain.

### 2.4 Climate-growth relationships

Analysis of variance (ANOVA) and Tukey’s HSD test were used to test the difference in xylogenesis between years. We assessed the relationships between monthly climate data and the phases of xylem phenology using Partial Least Squares regression (PLS). PLS is suitable to mitigate the multicollinearity among variables (Abdi, 2010; Rosipal and Krämer, 2005), mainly when the number of predictors is higher than the number of observations. The coefficients of the first component, which explains most of the variance, were further used to test the sensitivity of the effect of temperatures on xylogenesis by running the model at different monthly temperatures and comparing the corresponding outputs for each scenario.

PLS model was applied to CMIP6 grids to simulate the effect of climate scenarios on xylogenesis. Onset and ending of growth and cell production were simulated for 1981–2010 and 2051–2080, thus obtaining the variation among periods. Spatial analyses and statistics were performed with QGIS version 3.38, and the packages “pls”, “raster” and “terra” in R version 4.3.1.

## 3 Results

### 3.1 Climatic conditions

**Figure 1:**
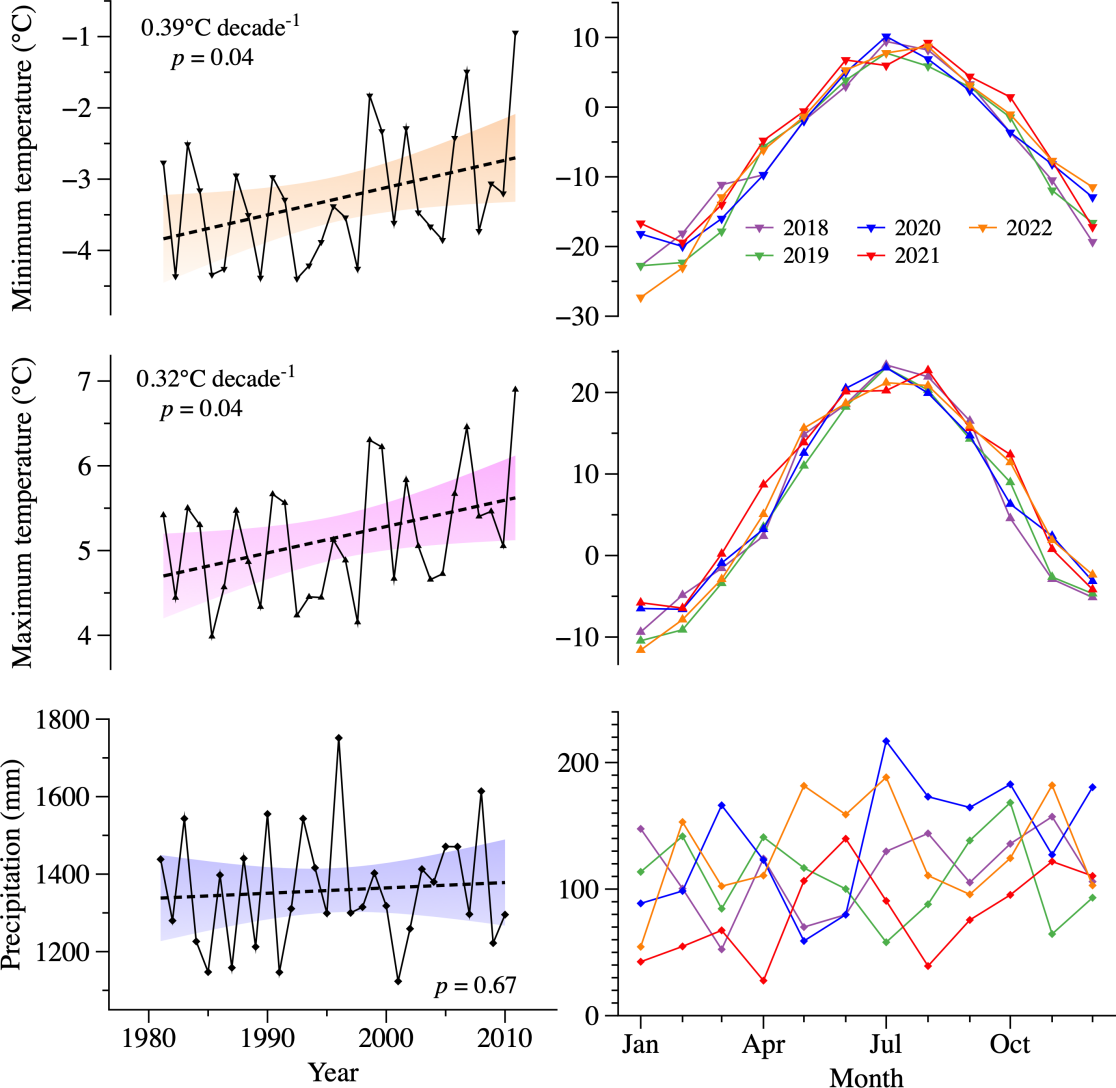
Climate condition in the study region. Annual minimum and maximum temperatures, as well as precipitation extracted from ERA5 during 1981–2010 are shown with black lines. Their respective linear trend is shown with dashed lines and the corresponding 95% confidence level is indicated by the filled area. Monthly temperature and precipitation data observed for each sampled year at the Montmorency site is indicated by the different colored lines.

According to the weather station data located in the study area, the average minimum, and maximum temperatures during 2018–2022 were -5.6 and 6.9°C, respectively. The year 2021 was the warmest, with maximum temperatures exceeding 0°C in March (**Supplementary Table S1**; **Figure 1**). In contrast, 2019 was the coldest year. The differences in annual maximum and minimum temperatures between 2021 and 2019 amounted to 2.4 and 1.8°C, respectively. On average, annual precipitation was 1371 mm. The lowest precipitations were recorded in 2021 (972 mm), particularly during the winter and early spring from January to April (192 mm).

A strong agreement was observed between the weather station data and ERA5 data during 1981–2010 (**Supplementary Figure S1**), with correlations reaching 0.88, 0.95 and 0.52 for the annual minimum temperature, maximum temperature, and precipitation, respectively (*p* < 0.05). The lower correlation for precipitation can be attributed to local factors (e.g., topography), the inherent spatial variability of precipitation, and the spatial resolution limitations of ERA5. The analysis of annual trends from 1981 to 2010 revealed an increasing pattern in both the mean annual minimum and maximum temperatures, with slopes of 0.39 and 0.32°C per decade, respectively (*p* < 0.05, **Figure 1**). In particular, autumn minimum and maximum temperatures indicated significant warming trends, with slopes reaching 0.57 and 0.47°C decade^-1^, respectively (**Supplementary Figure S2**). The slopes were not significant in spring, summer, and winter (*p* > 0.05). No significant linear trend was detected for the annual and seasonal precipitation (*p* > 0.05).

### 3.2 Characteristics of the xylogenesis

**Figure 2:**
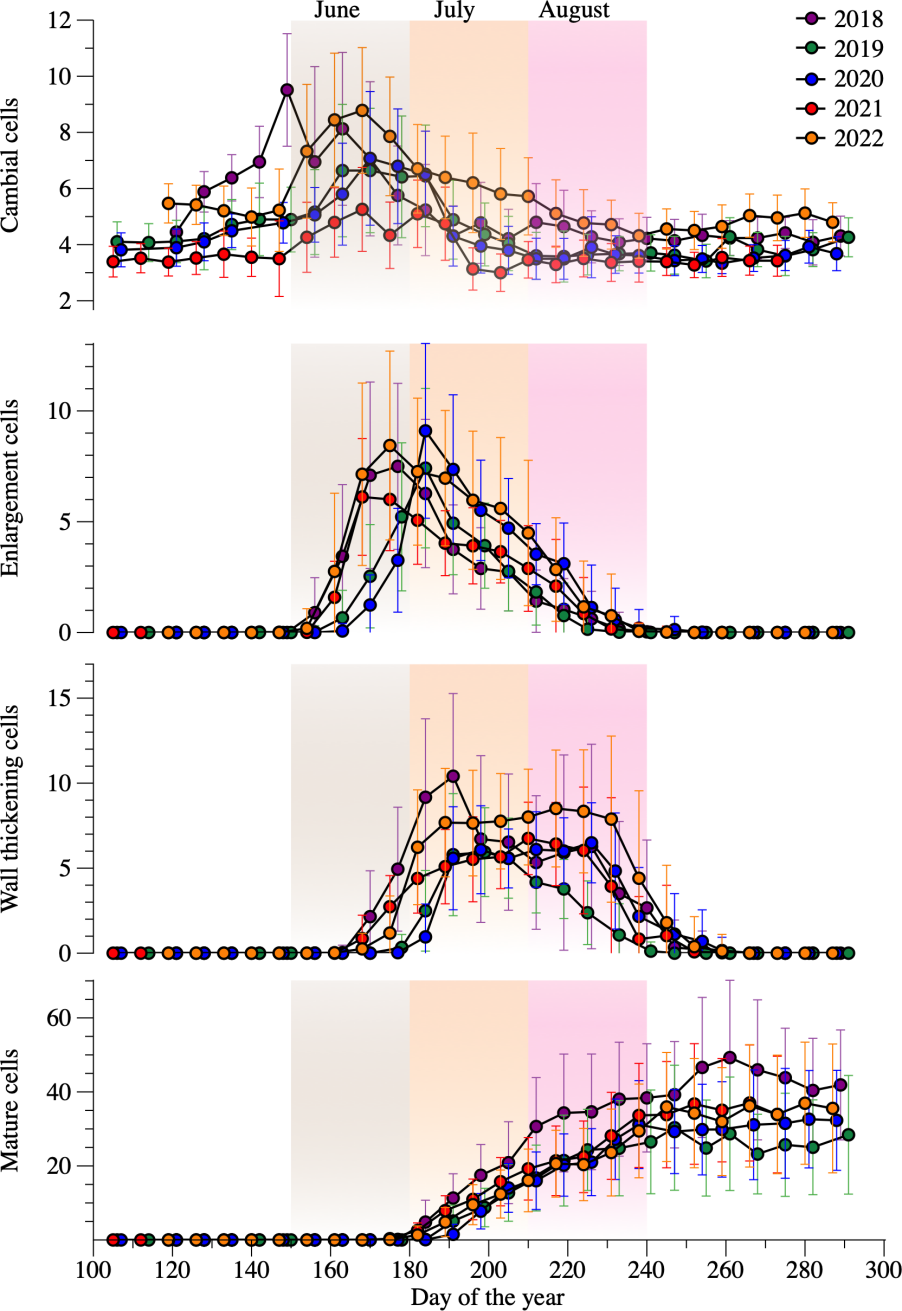
Evolution of each phenological phase between 2018–2022 period. Dots and bars represent average number of cells and standard deviations among the sampled trees for each year. The shaded areas of light gray, orange and magenta represent June, July, and August, respectively.

The dormant cambium was typically composed of 3–5 closely spaced cells. In spring and early summer, the cambial cells increased up to 21 (**Figure 2**). The earliest onset of enlargement occurred in 2021 on DOY 152. The latest occurred in 2019 on DOY 167. Overall, 11%, 87% and 2% of the sampled trees started enlargement in May, June, and July, respectively (**Supplementary Figure S3**). Nearly 96% of trees completed enlargement in the period between DOY 190 and 240. The duration of cell enlargement was < 30 days for 4% of trees, between 30 and 60 days for 55%, and > 60 days for 41%. Cell wall thickening and lignification started on average 12 days after the beginning of cell enlargement (**Figure 2**), with 82% of trees starting this phase in June. The ending of cell wall thickening spanned over two months, ranging from DOY 204 to 278, with 72% of trees ending lignification in August. On average, this developmental phase lasted approximately two months, more precisely 59 days. The longest and shortest duration of cell wall thickening occurred in 2018 (75 days) and 2019 (49 days), respectively. The first mature cell was observed at the beginning of July. The duration of xylem formation varied from 28–130 days, but lengths < 60 days and > 90 days accounted for only 14% and 17% of trees, respectively (**Supplementary Figure S4**). The growing season of 2019 was 14 days shorter compared to the other years.

Cell production ranged from 28 to 47 during the study years (**Supplementary Figure S4**), with a mean of 37 tracheids. The higher and lower growth was observed in 2018 and 2019, respectively. Mean cell production for the years 2020, 2021 and 2022 were 33, 37 and 36 tracheids, respectively. Overall, 13% of the trees produced < 30 cells, 69% produced 30 to 60 cells, and 18% produced > 60 cells.

### 3.3 Relationships between xylogenesis and climate

**Figure 3:**
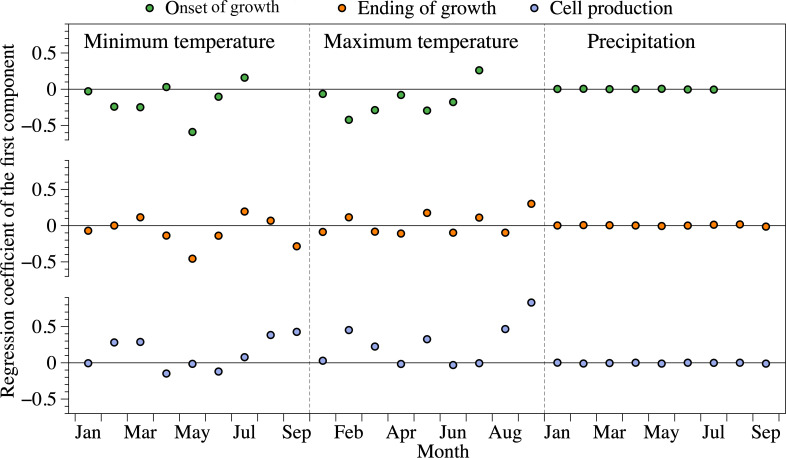
Regression coefficient between xylogenesis and climate data according to the first latent variable of the PLS. The climate variables were calculated from January to September for the ending of growth and cell production, while that was conducted from January to July for the onset of growth. Onset of growth indicates onset of enlargement, ending of growth means ending of cell wall lignification.

The first component factor of the PLS regression explained 93, 96 and 99% of the variability for the onset of enlargement, ending of lignification and cell production, respectively. These high percentages reflect a good fit of the PLS model and indicate that the first component explains most of the variability of the independent variables. According to the PLS correlation coefficients, negative relationships were detected between the onset of cell enlargement and minimum temperature in winter and spring. Maximum temperature from January to June was also negatively correlated with the onset of growth (**Figure 3**), with the lower correlation being observed in February (r = -0.42). The highest negative correlation was detected between the ending of cell wall lignification and May minimum temperature (r = -0.46). Cell production was positively correlated with minimum and maximum temperatures in February, March, and August. The correlation was close to zero between monthly precipitation from January to September and xylogenesis or cell production.

By running the PLS model under increasing monthly temperatures, we detected a gradual advancement of the beginning of xylogenesis (**Supplementary Figure S5**). In particular, increases in minimum temperatures resulted in a greater advancement than increases in maximum temperatures, particularly during spring (March to May). An increase of 1°C in maximum and minimum temperature advanced the onset of enlargement by 0.88 and 0.99 days, respectively. The impact of maximum and minimum temperature on the ending of xylogenesis was 0.07 and 0.91 days °C^-1^. An increase in minimum temperature during summer and autumn could delay the ending of xylogenesis by 0.55–0.71 days °C^-1^, while the effects of maximum temperature were lower, being 0.11–0.21 days °C^-1^. According to our simulations, higher temperatures were more likely to affect the onset of xylogenesis than the ending. Cell production demonstrated a stronger response to the minimum temperature of the previous autumn (0.62 cell °C^-1^), compared to the other seasons (0.34–0.38 cell °C^-1^).

### 3.4 Mapping xylogenesis under future temperature condition

**Figure 4:**
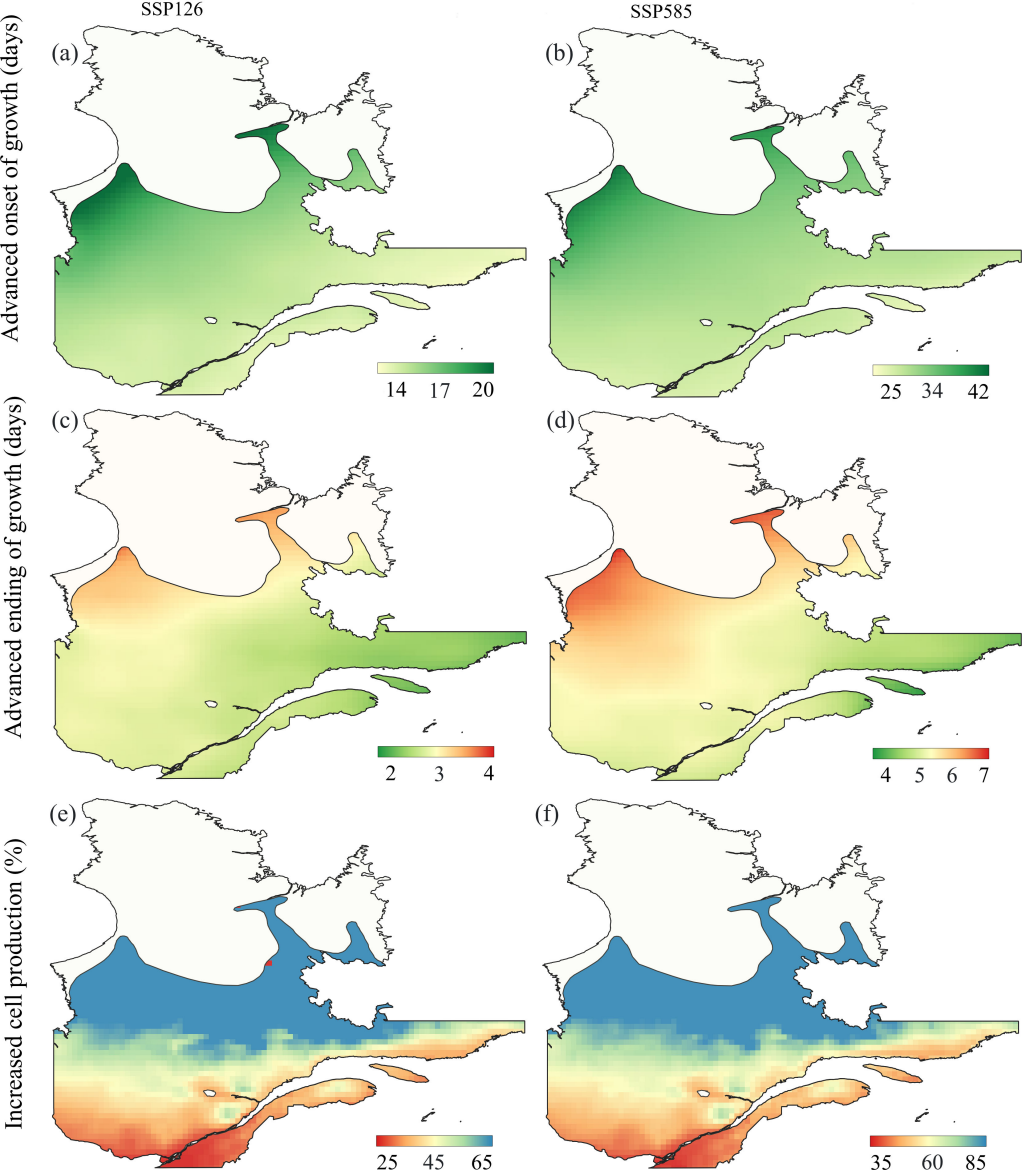
Averaged prediction for the onset of growth [days, **(a, b)**], ending of growth [days, **(c, d)**] and cell production [%, **(e, f)**] for the period 2051–2080, compared to the reference period 1981–2020, based on the two different scenarios (ssp126 and ssp585) from the CMIP6 simulation. The prediction was conducted on the balsam fir spatial range in Québec.

According to the most optimistic scenario of ssp126, the onset of enlargement could advance by 14–20 days during 2051–2080 compared to the reference period (**Figure 4a**). The growth reactivation could advance by 25–42 days under the ssp585 scenario (**Figure 4b**). The largest changes in the onset of growth were observed in the north and west compared to the south and east. The changes predicted for the ending of cell wall lignification were within one week (2–7 days) under both scenarios (**Figures 4c, d**). Hence, compared to the starting dates of growth, the ending could be less affected by the future warming. In contrast, cell production could increase by approximately 25–65% under the ssp126 scenario (**Figure 4e**) and 35–85% under the ssp585 scenario (**Figure 4f**). The increase in cell production followed a latitudinal gradient, with the higher changes being estimated at the northern region of the boreal forest.

## 4 Discussion

In this study, we investigated wood formation in balsam fir and its relationship with climate data over five years from 2018 to 2022, within the boreal forest of Québec, Canada, using an extensive microcore sampling. Our results support the initial hypothesis that warmer temperatures advance the onset of enlargement and enhance cell production. Our second assumption, proposing that warming has a stronger impact on growth reactivation compared with growth cessation, was also validated. These findings provide valuable insights into assessing the sensitivity of balsam fir to varying weather conditions, thus enhancing our understanding of the factors influencing the growth of the boreal forest under current and future climatic conditions.

### 4.1 Xylogenesis of balsam fir

Our study revealed significant annual and seasonal variations in cambial cells and xylem phenology in balsam fir from 2018 to 2022. The fluctuations in number of cambial cells, which peak in June and decline from July onward, is consistent with the typical growth pattern observed in temperate and boreal tree species (Chen et al., 2019; Huang et al., 2014, 2020; Rossi et al., 2014; Silvestro et al., 2024). In 96% of trees, cell enlargements ended between DOY 190 and 240 during the five study years, which is consistent with other species in the boreal environment (Huang et al., 2011; Zeng et al., 2022; Zhai et al., 2012; Silvestro et al., 2024). Secondary cell wall lignification was completed on 72% of trees in August, a result that is consistent with the need for the newly formed xylem to complete lignification before winter dormancy to provide mechanical support and water transport capacity for the upcoming year. The linear relationship between mean annual temperature and timings of xylem phenology proposed for conifers in a cold climate (Rossi et al., 2016) also applies to the balsam fir in our study region. Our results further showed an important phenological variability among trees within the same population, as previously observed by Silvestro et al. (2022) at intra-annual scale, e.g., the range of the mean starting dates of cell enlargement across the years varied from DOY 152 to 167. This variability is a crucial factor to consider in modeling wood formation to avoid biased conclusions. However, further studies are necessary to clearly highlight the factors underlying this variability.

### 4.2 Effects of climatic conditions on xylogenesis

Air temperature affects the onset more than the cessation of wood formation in balsam fir. Spring temperatures have previously been identified as a primary driver of growth reactivation in temperate and boreal forests (Delpierre et al., 2019; Huang et al., 2020; Rossi et al., 2016). After accumulating enough winter chilling and broken endodormancy, spring temperatures contribute to the accumulation of forcing units, ultimately triggering the end of ecodormacy and reactivation of growth processes (Begum et al., 2018; Fu et al., 2015; Huang et al., 2023; Perry, 1971). Studies across various regions showed that warmer spring temperatures lead to an earlier initiation of cambial activity, advancing the onset of xylogenesis (Deslauriers et al., 2008; Saderi et al., 2019). At our study site, the coldest mean minimum temperature in March (-17.8°C), recorded in 2019, resulted in the most delayed onset of enlargement (DOY 167) across the study years. Conversely, the warmer temperature recorded in March 2021 (-14.0°C) advanced the mean starting dates by nearly 15 days, compared to 2019. Overall, the strong influence of spring temperatures on the start of the growing season highlights the relationship between climate and xylem phenology in the boreal forests, making these ecosystems vulnerable to shifts in seasonal climate.

March minimum temperature in 2021 also resulted in increased cell production (37 tracheids), compared to the colder conditions observed in 2019 (28 tracheids). In ecosystems where temperature is a limiting factor, warmer spring conditions can create a favorable environment for trees to boost metabolic activity and extend the growing season, thereby supporting increased cell division in the cambium. Consistently, previous studies have shown that a longer growing season corresponds to greater xylem cell production in balsam fir (Silvestro et al., 2022) and that, in general, warmer temperatures increase cell production in black spruce in eastern boreal forests (Rossi et al., 2014; Zeng et al., 2022). Moreover, we observed a prominent positive correlation between the minimum temperature of the previous autumn and cell production. Warmer autumns can extend the photosynthetic period (Chang et al., 2016; Stinziano et al., 2015) and stimulate root growth and mycorrhizal activity, helping the trees to absorb more nutrients from the soil before entering dormancy (Domisch et al., 2002). These additional nutrients could be stored and utilized in the following spring when cell production resumes, contributing to a stronger growth response.

We were unable to find significant effects of precipitation on phenology and cell production, although xylogenesis in balsam fir has been demonstrated to be sensitive to water shortage in rain exclusion treatments (D’Orangeville et al., 2013a). During 1981–2010, significant warming trends were observed in the autumn maximum and minimum temperatures. This change may have enhanced cell production in balsam fir. If the warming trend continues in the future, we could expect that additional changes in the growth patterns could modify wood anatomical features in balsam fir (Silvestro et al., 2023), possibly leading to modifications in biomass accumulation at the stand level.

Compared to the onset of growth, the ending of growth is less affected by temperature variables as indicated by their weaker correlations in our PLS model. Other factors, e.g., photoperiod, may contribute to the ending of growth (Cooke et al., 2012; Duchesne et al., 2012; Perrin et al., 2017). Moreover, a relationship exists between the timing of onset and ending of wood formation. In balsam fir, an earlier onset of wood formation is associated with a later ending of the growing season (Silvestro et al., 2022, 2023). Moreover, a higher production of xylem cells in differentiation during the early growth phase can result in a delayed end to the growing season (Lupi et al., 2010). The relationships among phenological events should be investigated more deeply to quantify the link between timings and amount of wood growth.

Our results suggest that xylogenesis is more responsive to minimum than to maximum temperatures. Wood formation in conifers can only occur when a minimum temperature of 4-5°C is reached (Rossi et al., 2008). Moreover, xylogenesis typically occurs during the night and early morning hours (Zweifel et al., 2021) when temperatures are at their lowest. This suggests that minimum temperatures directly impact the cellular processes involved in wood formation, such as cell division and elongation. Indeed, minimum air temperature was identified as the dominant climatic variable for xylem growth of Smith fir (Li et al., 2017). In this context, high maximum daytime temperatures have been shown to act as a potential stressor during the growing season, rather than at its onset (Isabelle et al., 2021).

### 4.3 Response of xylogenesis to future warming

Predictive modeling under climate scenarios indicates that the onset of cell enlargement could advance by 14–42 days over the period 2051–2080, compared to the mean values during the period of reference (1981–2010). This advancement is more pronounced in the north and west of balsam fir distribution, highlighting some regional differences in the effects of climate variations across our study area. An anticipated onset of the growing season has also been predicted for black spruce under different warming scenarios (Rossi et al., 2011). The ending of xylogenesis at our study site was projected to remain relatively stable, suggesting that the overall growing season may lengthen primarily through an earlier initiation rather than a delayed cessation. Based on our model, cell production is expected to increase under both minor and severe warming scenarios, with potential rises of 25–85%. Localized warming experiment could also increase cell production in the stem of *Picea abies* (Gričar et al., 2006). These findings suggest that trees from colder climates could directly benefit from warmer temperatures, although the water stress induced by higher temperatures may reduce the potential benefits of warming.

Our analysis reflects the influence of temperature, and the impact of precipitation, particularly in sites where water is more limiting, may be underestimated. We should also note that while moderate warming can enhance growth in terms of number of xylem cells produced, excessive heat and prolonged droughts associated with climate change could trigger new stressing conditions for balsam fir. This stress can reduce the growth rate, make the trees more susceptible to pests and diseases, and potentially lead to higher mortality rates in extreme cases. The increased frequency and intensity of droughts in the future may have negative effects on the duration of xylogenesis and the production of xylem cells in balsam fir (D’Orangeville et al., 2013a). Results also showed that balsam fir was projected to decline under climate change in the Acadian Forest region of eastern Canada (Collier et al., 2024). Hence, interactive modeling including precipitation and wood formation dynamics would provide more complete projections of the growth under climate change, as studies have shown that the different climate conditions can drive variations in gross primary productivity and woody biomass in the boreal forest (Puchi et al., 2023, 2024).

## 5 Conclusion

In conclusion, by deepening our understanding of balsam fir sensitivity to temperature variability, this research reveals potential shifts in growth dynamics that could affect ecosystem services, including carbon sequestration. Future research should focus on the role of precipitation, which, while not significantly correlated with xylogenesis in this study, may influence growth under more water-limited conditions. Considering our prediction of wood formation is based on one study site, long-term monitoring and modeling at multiple sites remain essential to refine predictions and inform conservation practices under future climate change scenarios. Evaluating the extent to which the growth of balsam fir may benefit from warming conditions will be an ongoing research priority for the boreal forest of Eastern Canada.

## Data Availability

The original contributions presented in the study are included in the article/**Supplementary Material**. Further inquiries can be directed to the corresponding author.
