# Supplementary material for: Xylogenesis under future climates: enhanced growth of balsam fir in a warming boreal forest
Source: Front Plant Sci. 2025 Jul 10;16:1563051. doi: 10.3389/fpls.2025.1563051 (PMC12286939; doi:10.3389/fpls.2025.1563051)
Supplement: Supplementary file 1 [file DataSheet1.pdf]

Xylogenesis under future climates: enhanced growth of balsam fir in a warming boreal forest

Minhui He[1,2], Jean-Daniel Sylvain[3,4], Roberto Silvestro[1], Guillaume Drolet[3], Richard Arsenault[4], Sergio Rossi[1]

[1]Laboratoire sur les écosystèmes terrestres boréaux, Département des Sciences Fondamentales, Université du Québec à Chicoutimi, 555 boulevard de l'Université, Chicoutimi (Québec), G7H2B1, Canada
[2]Centre de Géomatique du Québec, Chicoutimi (Québec), G7H 1Z6, Canada
[3]Direction de la recherche forestière, Ministère des Ressources naturelles et des Forêts, Québec, G1P3W8, Canada
[4]Hydrology, Climate and Climate Change Laboratory, École de Technologie Supérieure, 1100 Notre-Dame W., Montréal, H3C1K3, Canada

Supplementary material

Table S1 Detailed information of annual (January–December) climate data and the balsam fir trees used for the monitoring of xylogenesis in the five years at the Montmorency Forest, Québec, Canada.

Year	2018	2019	2020	2021	2022
Number of trees	160	162	162	160	167
Minimum temperature (°C)	-6.11	-6.66	-5.53	-4.38	-5.51
Maximum temperature (°C)	6.53	5.76	7.13	8.18	7.16
Precipitation (mm)	1352	1308	1660	972	1565

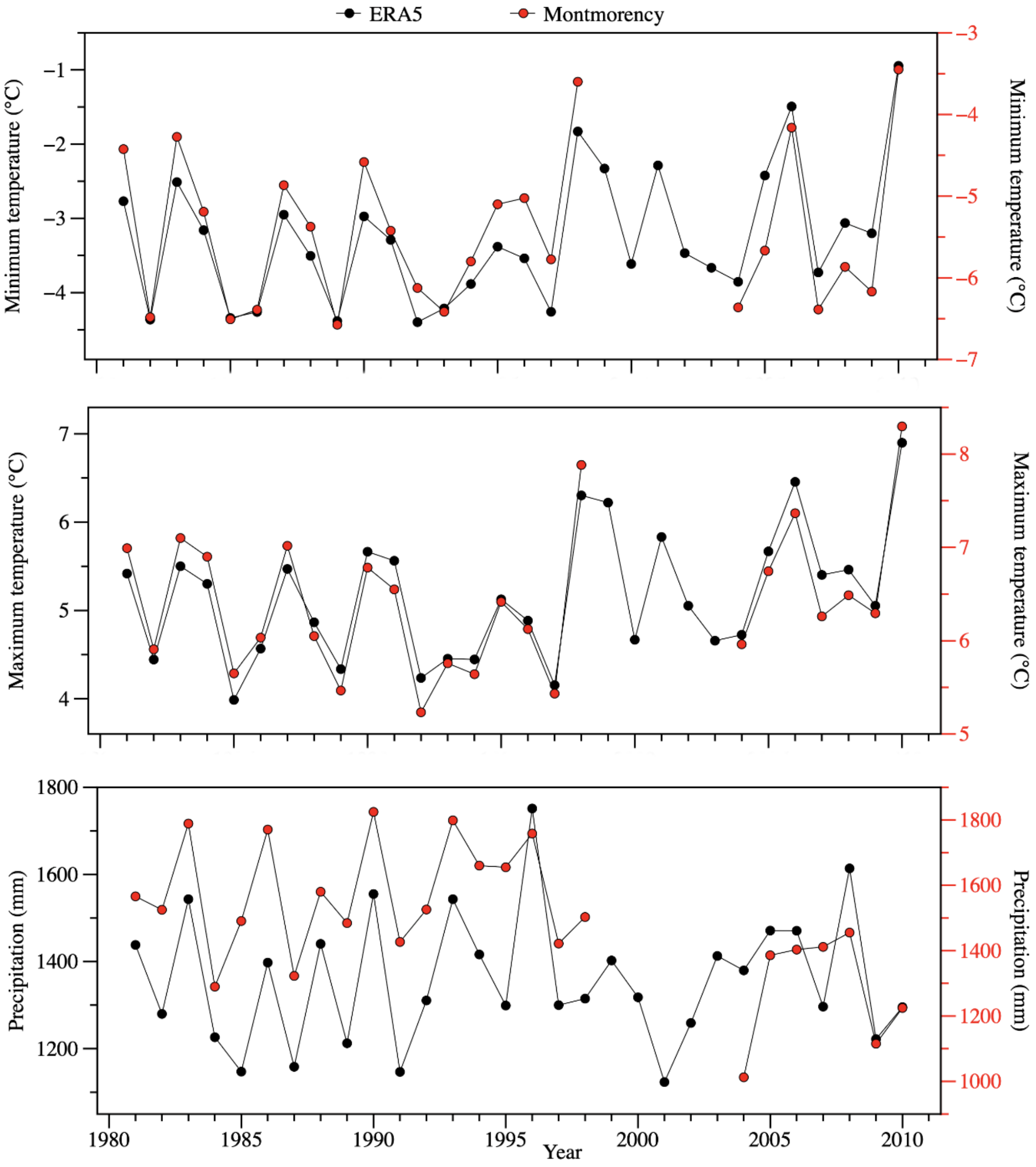

Fig. S1 Annual minimum temperature, maximum temperature, and precipitation data extracted from ERA5 and Montmorency weather station during the period 1981–2010.

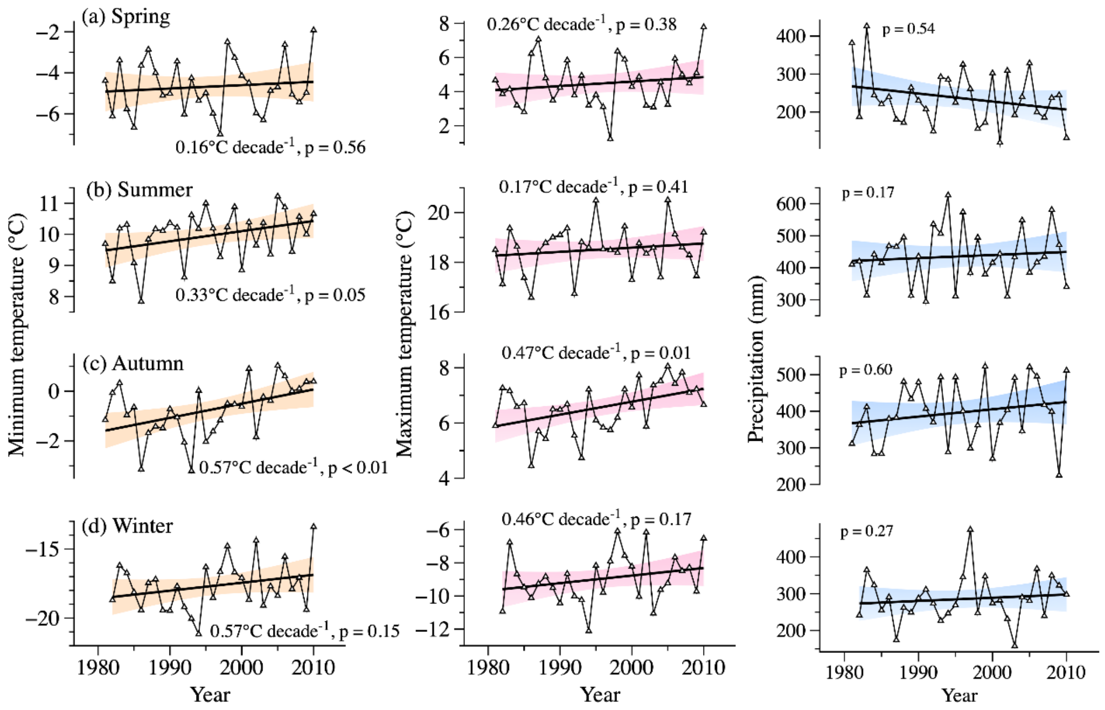

Fig. S2 Seasonal ERA5 temperature and precipitation condition from 1981 to 2010 in the study region. Spring: March–May; Summer: June–August; Autumn: September–November; Winter: December–February. The long-term linear trend is shown with black lines and their corresponding 95% confidence level is indicated by the filled area. Autumn temperature significantly increased over the past 30 years ($p < 0.05$).

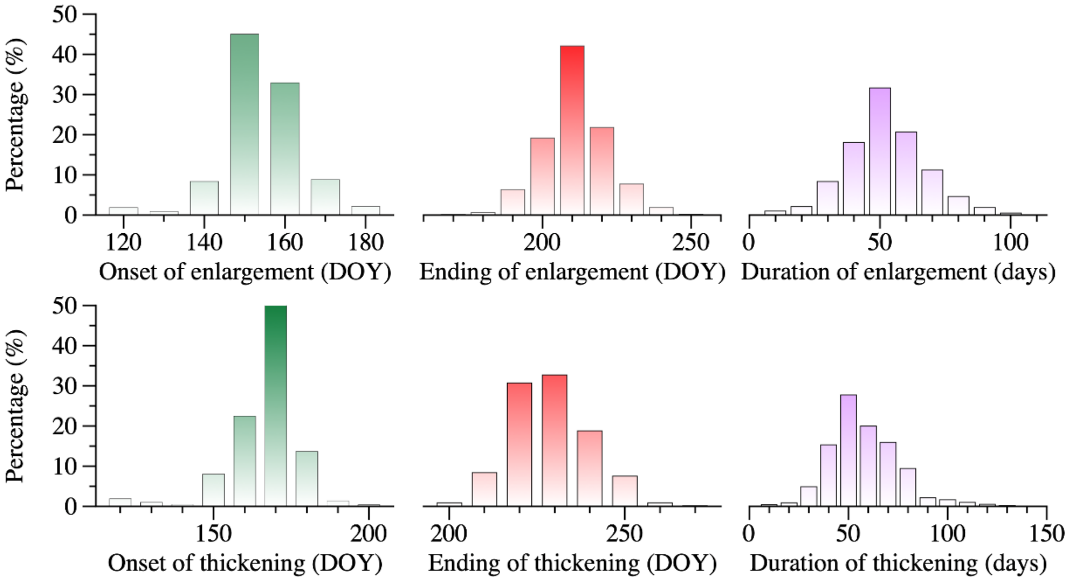

Fig. S3 Characteristics of the onset and ending dates of wood formation based on the five years data in the study region.

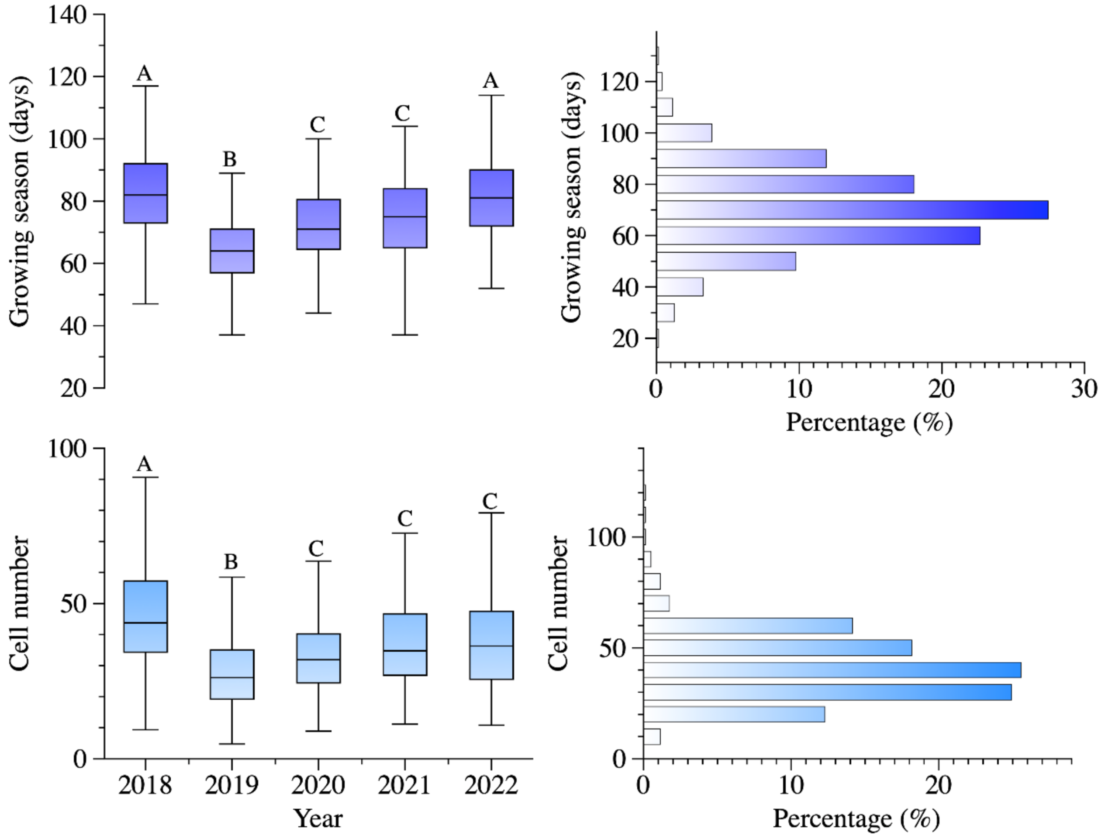

Fig. S4 Distribution of duration of the growing season and cell production across years. The letters A, B and C indicate significant differences between the means of each group at the $p < 0.05$ level.

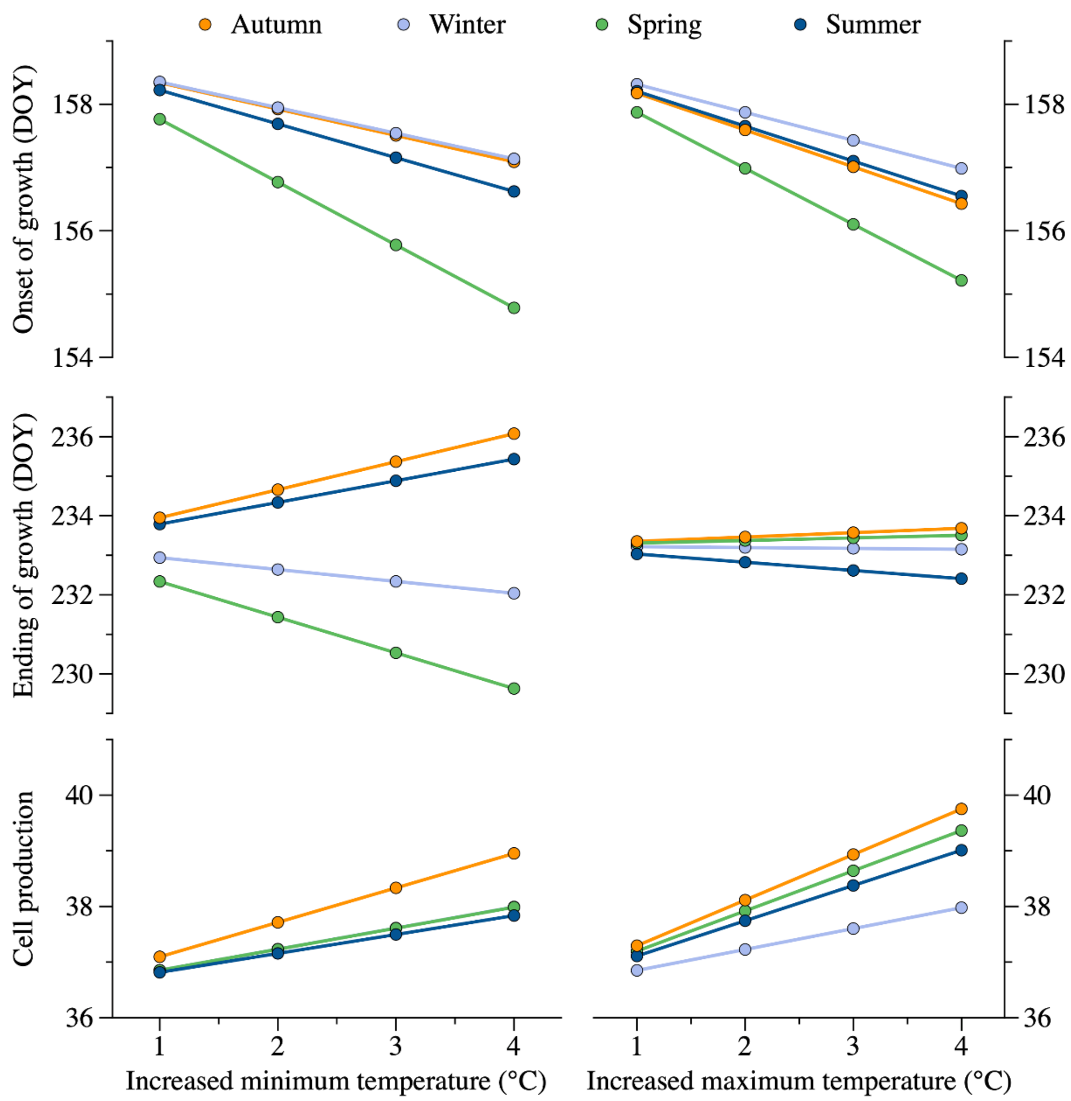

Fig. S5 Response of the onset of growth, ending of growth and cell production to seasonal minimum and maximum temperature. This prediction was conducted by PLS. Autumn: previous autumn from September to November; Winter: December–February; Spring: March–May: Summer: June–August.

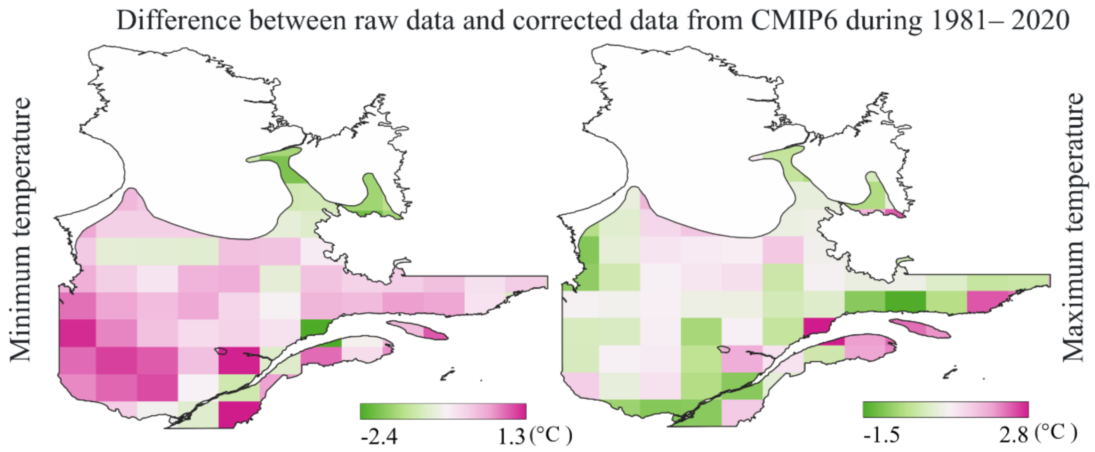

Fig. S6 Averaged temperature (January–September) difference between raw data and bias-corrected prediction data from CMIP6 during the period 1981–2010.
